# Examining the optimum panel pillar dimension in longwall mining considering stress distribution

**DOI:** 10.1038/s41598-024-57579-w

**Published:** 2024-03-22

**Authors:** Mustafa Emre Yetkin, Muharrem Kemal Ozfirat, Turgay Onargan

**Affiliations:** Mining Engineering Department, Engineering Faculty, Dokuz Eylul University, Buca, Izmir, Turkey

**Keywords:** Longwall mining, Pillar, Numerical analysis, Stress distribution, Environmental sciences, Engineering

## Abstract

Longwall mining method is widely used for underground coal production in the world. Additional stresses occur surrounding the longwall during underground mining. Stresses occurring surrounding the longwall are investigated by many researchers for years. How these stresses affect longwall production, gob, main gate, tailgate and main haulage road has been always an important issue. In this study, the effect of the safety pillar left at the end of the panel on the main haulage road is investigated. For this purpose, 6 models with different pillar distances are created and the stresses occurring in the main haulage road, tailgate and main gate at different pillar distances are examined. It has been demonstrated with numerical models that the optimum pillar distance according to these stress conditions does not damage the main haulage road, tailgate and main gate. In addition, the pillar distance of 10 m gives maximum coal recovery efficiency, and it has been shown by numerical models that the stresses occurring in the main haulage road, main gate and tailgate are not damaging to these galleries.

## Introduction

In Turkey and globally, the extraction of coal seams is frequently conducted through the utilization of longwall method. According to^1^, longwall mining is an exploitation method commonly applied in flat deposits, where the face is established for mineral extraction. Nowadays, longwalls are designed to be approximately 300 m in width and 1–2 km in length, depending on the seam structure. The coal seam prepared for production becomes ready for extraction through the combination of galleries, known as the gate road and road ways, which are opened parallel to the coal seam. The gate roads play a crucial role in the layout of longwall mining as they serve as the exclusive escape and access routes to the longwall face^2–4^. Today, lignite mining heads for deeper mining all around the world. Lignite mining in countries such as Turkey, China, and Australia will be performed at deeper levels from now on. In this case, the importance of stress–strain and numerical modeling studies in the literature is increasing. Examining the previous numerical modeling studies, it is seen that there are several studies carried out. Reference^5^ has studied the same coal field. They have revealed that maximum vertical abutment stresses were formed at a distance of 7 m in front of the face in the numerical modeling they made. With a combination of analytical, observational and empirical methods, “Ref.^6^ developed a new cavability assessment criterion for longwall roof strata cavability, and they assisted in stress modeling”. “Reference^7^ investigated the effect of rock bolt penetration on roof loads to ensure the stability of the tailgate-main gate in the longwall production method”. “Reference^8^ identified the relative magnitude of horizontal stress change below a series of parallel longwall panels as a consequence of multi-seam mining with finite element modeling”. “Reference^9^ made numerical modeling for the loads in critical regions determined on the longwall panel and for the forming zone of broken rock rubble, called gob. “Reference^10^ made stress modeling of bauxite seam in the longwall, added discontinuities to the model, and they revealed that the pillar left between two panels is sufficient by using numerical modeling”. “Reference^11^ showed the effect of discontinuities on the stresses on the pillar in the longwall top coal caving (LTCC) with a numerical model”. “Reference^12^ calculated safety factors according to the pillar size to be left between two panels by numerical modeling”. “Reference^13^ revealed that additional stresses occur on the longwall panel after the face advances 120 m”. “Reference^14^ carried out studies on stress numerical modeling on longwall chain pillars with drill techniques”. “Reference^15^ tried to model the maximum convergence values from roof to floor in a mechanized longwall under poor main roof conditions”. Also, numerical methods provide powerful tools for the analysis and design of LTCC mining operation systems with complex mining conditions^5,16–30^. “Reference^31,32^ studied mining cribs models in underground mines and aims to enhance stability, minimize resource wastage, and potentially improve cost-efficiency using numerical analysis, while also emphasizing the importance of proper design, durability, safety considerations, and compliance with regulations in implementing this alternative support system in underground mines.”

In this study, the optimum dimensions of the pillar to prevent the main road and panel tailgate-main gates from being affected by production-induced stresses towards the end of the panel in the production of longwall are investigated through numerical modeling studies. In the models, pillar distances from 10 to 60 m are modeled. In the numerical modeling, the lowest mean stress occurring in the main road is observed in the pillar distance of 10 m with 8.1155 MPa. In addition, when pillar distance of 10 m is left, the coal recovery efficiency in the panel reaches the highest value. Therefore, it is calculated that pillar distance of 10 m does not reach a stress value that will affect the production on the main road and tailgate-main gates.

## Boundary conditions and rock mass calculations

In engineering applications, defining the physical and mechanical properties of the rock material as well as the rock mass behavior is very important in roof stress–strain analysis and support design.

**Figure 1:**
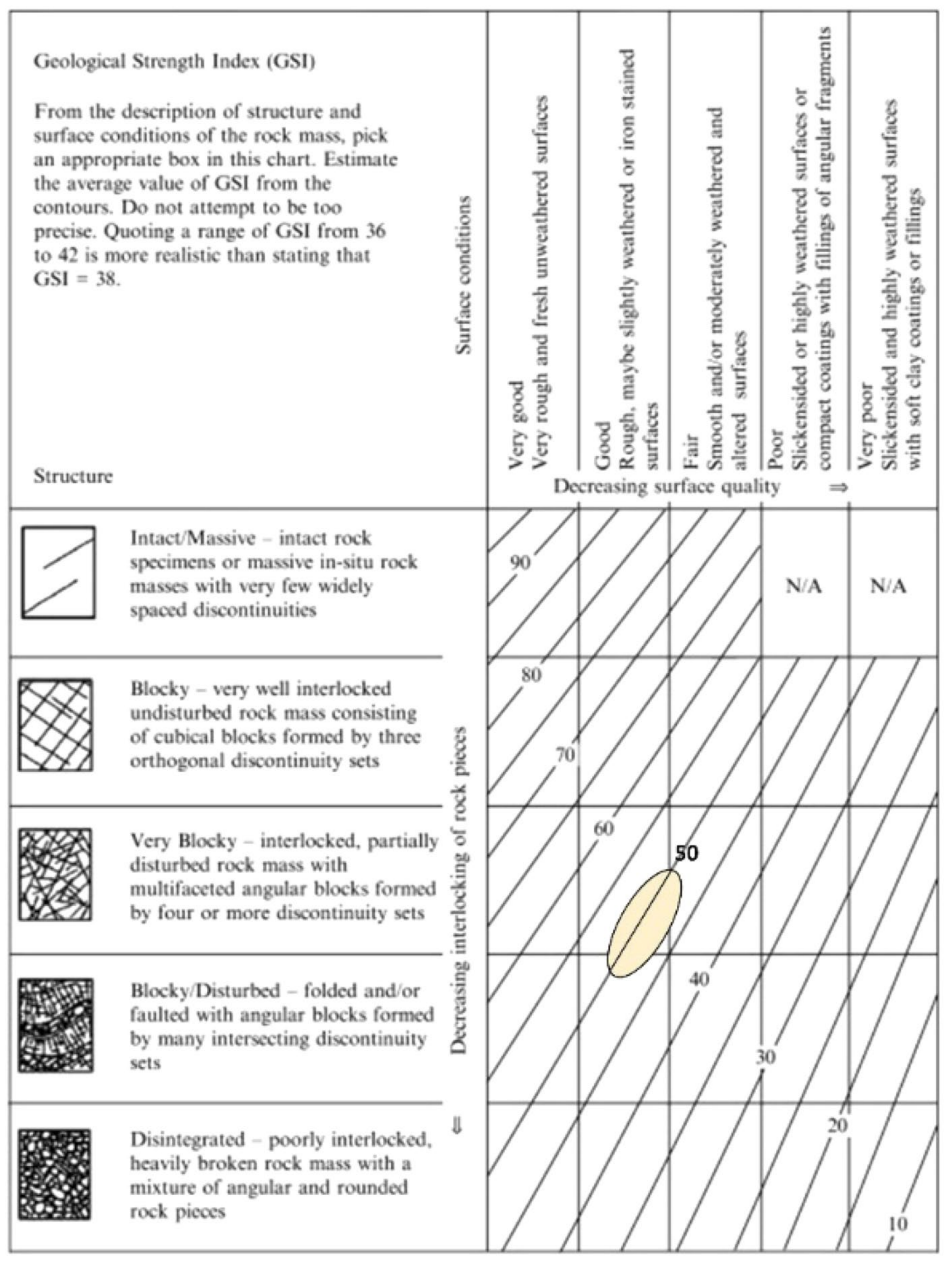
Characterization of Marl and Coal rock mass classifications^33^.

Reference^33^ introduced the geological strength index (GSI), both for hard and weak rock masses. Experienced engineers and miners generally prefer simple, fast, but reliable classification which is based on visual control of geological conditions. Reference^33^ proposed such a practical classification for estimating GSI based on only visual inspection (Fig. 1). Based on the real rock structure classification and the discontinuity surface condition, a block in the 5 × 5 matrix of Fig. 1 can be found and the corresponding GSI value can be read from the figure. According to^33^, a range of values of GSI should be estimated in preference to a single value.

The Hoek–Brown failure criterion is a widely accepted approach for predicting the relationship between principal stresses at the point of failure in a rock mass. Through trial-and-error methodology, it was determined that the relationship between the major principal stress and the minor principal stress is curved. The failure criterion establishes a connection between the major principal stress (σ_1_) and the minor principal stress (σ_3_) at the point of failure. The equation describing this criterion is provided below as Eq. (1).

Generalized Hoek–Brown criterion is presented in Eq. (1).

1 $${\sigma }_{1}={\sigma }_{3}+{\sigma }_{ci}{\left({m}_{b}\frac{{\sigma }_{3}}{{\sigma }_{ci}}+s\right)}^{a}$$

In the Hoek–Brown failure criterion:

σ_1_ and σ_3_ represent the maximum and minimum effective stresses at failure, respectively. mb is the value of the Hoek–Brown constant for the rock mass. s and a are constants that rely on the characteristics of the rock mass. σ_ci_ is the uniaxial compressive strength of the intact rock. The constant "s" is linked to the tensile strength between the blocks or grains within the rock mass and the degree of adherence of the rock material. In the case of the rock material, the typical value of "s" is 1. This value decreases from 1 to 0 in relation to the quality of the rock mass. The constant "mb" is subject to variation based on factors such as the specific type of rock in consideration.

Once the Geological Strength Index has been predicted, the parameters which describe the rock mass strength characteristics, are calculated as follows:

2 $${m}_{b}={m}_{i}exp\left(\frac{GSI-100}{28}\right)$$

For GSI > 25, i.e. rock masses of good to reasonable quality, the original Hoek–Brown criterion is applicable with

3 $$s=exp\left(\frac{GSI-100}{9}\right)$$

4 $$ {\text{a}} = \, 0.{5} $$

For GSI < 25, i.e. rock masses of very poor quality, the modified Hoek–Brown criterion applies with

5 $$ {\text{s}} = 0{\text{ and a }} = \, 0.{65 }{-} \, \left( {{\text{GSI}}/{2}00} \right) $$

For GSI = 25 the modified Hoek–Brown criterion applies with

6 $$ {\text{a}} = \, \left( {{125 }{-}{\text{ GSI}}} \right)/{2}00 {\text{a }} = \, 0.{5} $$

Laboratory experiments and field studies are carried out to find intact rock and rock mass properties. It is important to use rock mass properties instead of rock material properties in numerical modelling. That’s why, GSI classifications are made according to the^33^ as seen Fig. 1. Physical and mechanical properties of intact rock material are given in Tables 2 and 3.

Rock mass materials show elastic behaviour under low stress. As stress increases rock mass shows plastic and viscosity behaviour. The failure criterion is used to determine whether stress in the rock will lead to inelastic behaviour, including fracture in rock mass. For rocks under high stresses, plastic deformation occurs and brittle failure follows plastic deformation. The failure criterion is used to compute the plastic deformation. Two widely used failure plane criteria for rocks are the Mohr–Coulomb model and the Drucker-Prager model. The stress strain in the rock mass can be macro fractured into an elastic and plastic part. The elastic part of the strain can be computed from a linear elastic constitutive model.

**Figure 2:**
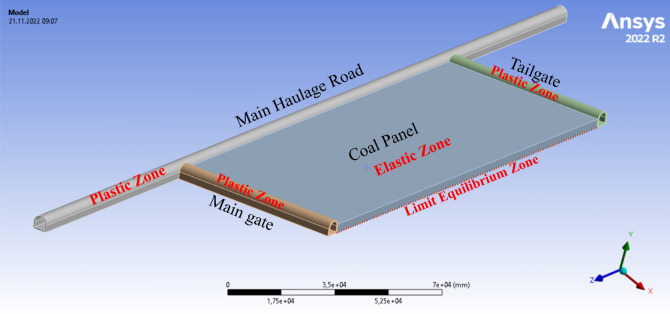
Boundary conditions in coal panel^41^.

Due to the complex distribution of the initial underground and mining stress fields, stress fields cannot be analyzed and described entirely. Thus, the following admissions are necessary in the model. With a continuous increase in the front abutment pressure, internal macro-cracks form in the coal panel, which then enters the limit-equilibrium state according to the^33^. In the coal panel, longwall face is accepted as limit equilibrium zone. Main and tailgate are accepted as plastic zone. The panel part to be left as pillar is modelled as elastic zone since it is not disturbed (Fig. 2).

Longwall modeling consists of three basic stages. These are;

1. Meshing
2. Definition of material behavior
3. Definition of boundary and initial condition

**Figure 3:**
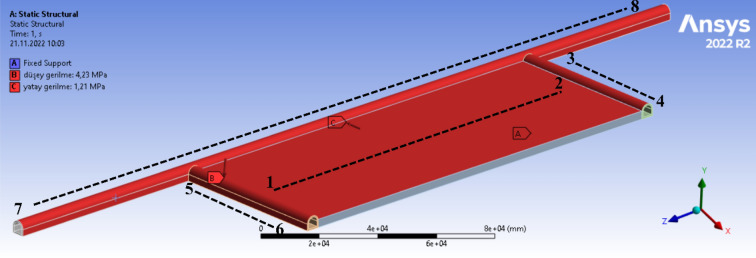
Boundary conditions of calculated area borders^41^.

Thanks to boundary and initial conditions, physical limits of the model and original conditions are implemented in the model. Boundary conditions applied in the model and stress values are given in Fig. 3 and Table 1.

**Table 1:** Boundary conditions stress values of the model.

Boundary	Vertical Stress (MPa)	Horizontal stress (MPa)
1–2	4.23	–
3–4	4.23	1.21
5–6	4.23	1.21
7–8	4.23	1.21

Considering the working depth, density values, and elasticity modulus of the rocks in the field (given in Table 3); the vertical and horizontal in-situ stresses that will occur on the panel, tailgate-main gate, and main haulage road are calculated using the equations given below^34^.

7 $$ \sigma_{{\text{h}}} = {\text{k }} \times \, \sigma_{{\text{v}}} $$

8 $$k=0.25+7\times E\times \left(0.001+\frac{1}{z}\right)$$

where, σ_V_: Vertical in-situ stress (MPa), σ_h_: Horizontal in-situ stress (MPa), k: The ratio of horizontal in-situ stress to vertical in-situ stress, E: Average modulus of elasticity of rock masses in vertical direction up to the worked depth (GPa) and z: Working depth (m).

By using Eqs. (7) and (8), at 200 m working depth, vertical and horizontal in-situ stresses are found as 4.23 MPa and 1.21 MPa, respectively.

Here, coal is extracted by retreating longwalls, employing top coal caving in longwall faces that are 3 m high, positioned at the bottom of the coal seam. The remaining 5 m thick top layer of the seam is subsequently caved and extracted through the use of a folding gob shield located at the rear of the shields. In the field, the daily progress rate averages 3.2 m.

## Calculation of in-situ stresses and creating models

In the present study^35^, solid modeling software is used and a longwall panel of 3 m’ seam thickness and 150 m’ length is modeled. In longwall mining, in order that additional stresses occurring in and around the longwall do not affect the main haulage road at the end of the panel produced, production is stopped at a certain distance to the end of the panel and a safety pillar is left for safety purposes.

**Figure 4:**
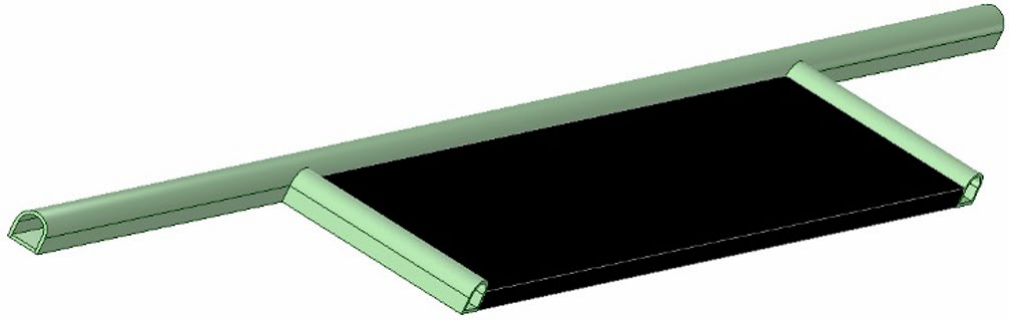
Longwalls modeled in different pillar distances^35^.

In the present study, a total of 6 longwall models are created with pillar distances of 60 m, 50 m, 40 m, 30 m, 20 m and 10 m respectively. Figure 4 shows the view of the modeled longwalls. Considering the panel dimensions, the areas used on the model for stress analysis are 9000 m^2^ for 60 m pillar distance, 7500 m^2^ for 50 m pillar distance, 6000 m^2^ for 40 m pillar distance, 4500 m^2^ for 30 m pillar distance, 3000 m^2^ for 20 m pillar distance and 1500 m^2^ for 10 m pillar distance.

**Figure 5:**
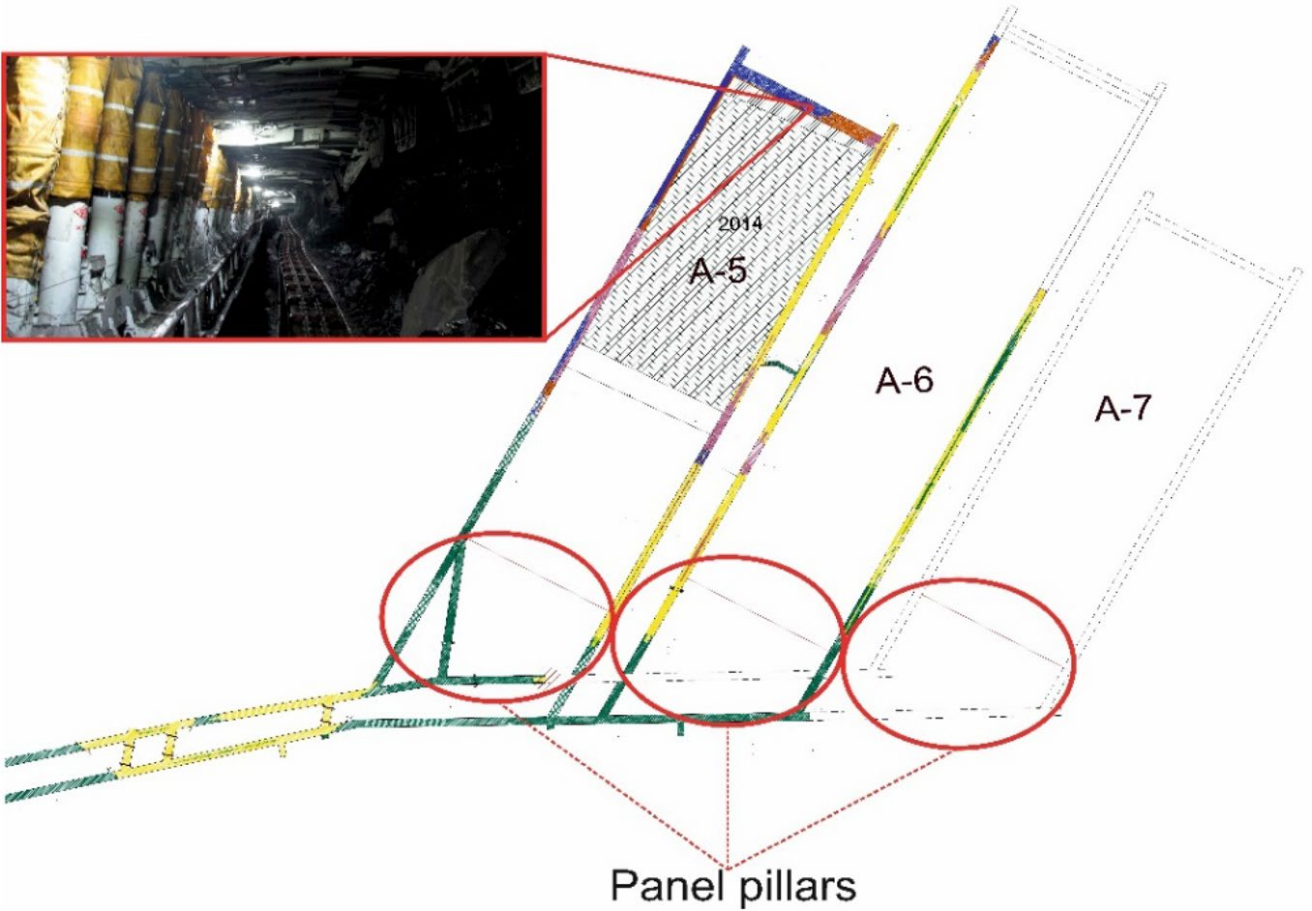
Panel plans and view of longwall (not to scale).

In modeling studies, the geomechanical parameters of the coal and surrounding rocks of the Tuncbilek region are taken into account. The mine depth is 200 m. Table 2 shows the geomechanical parameters of coal and surrounding rocks in the field. The data provided in Table 2 are actual results obtained from rock mechanics tests conducted on coal and surrounding rock samples taken from the field. In converting these data into rock mass data, the Generalized Hoek–Brown criterion given in Eq. (1) is used. Since the site is represented as a mass in modeling studies, the rock material properties given in Table 1 are transformed into rock mass data using^36^. The rock mass data used in models is given in Table 3. Figure 5 shows the plan views of the longwall panel and pillars in the worksite and the view of the longwall.

**Table 2:** Geomechanical parameters of coal and surrounding rocks^5,38–40^.

Formation	Density γ, MN/m^3^	Uniaxial compressive strength σ_c_, MPa	Modulus of elasticity Ei, MPa	Geological strength index (GSI)
Marl	0.022	16.10	2530	52
Coal	0.013	12.15	1748	47

**Table 3:** Rock mass data used in the model.

Formation	Density γ, MN/m^3^	Uniaxial compressive strength σ_c_, MPa	Modulus of elasticity Ei, MPa	Poisson’s ratio, υ
Marl	0.022	1.089	874.86	0.25
Coal	0.013	0.613	445.26	0.25

According to previous research, the gob zone in longwalls is categorized into three groups: new gob, compressed gob, and well-compressed gob. In their modeling study conducted in 2013, “Ref.^37^ assigned a Poisson's ratio of 0.25 to well-compressed gob, a Poisson's ratio of 0.30 to compressed gob and a Poisson's ratio of 0.40 to new gob. Similarly, they set the elasticity modulus of new gob to 100 MPa and elasticity modulus of compressed gob to 200 MPa, while the elasticity modulus of well-compressed gob is determined as 500 MPa”. As the longwall advances, the old gob zone becomes more compressed, influenced by the overlying strata. Theoretically, the internal friction angle of well-compressed gob decreases. This can be better understood by comparing the measured internal friction angle of a mass in its free state with the internal friction angle when the same mass is subjected to vertical compression. In the created model, the gob material is also divided into three groups using same strength parameters. Since gob material consists of fragmented material, the mass behavior in the model is selected as plastic.

**Figure 6:**
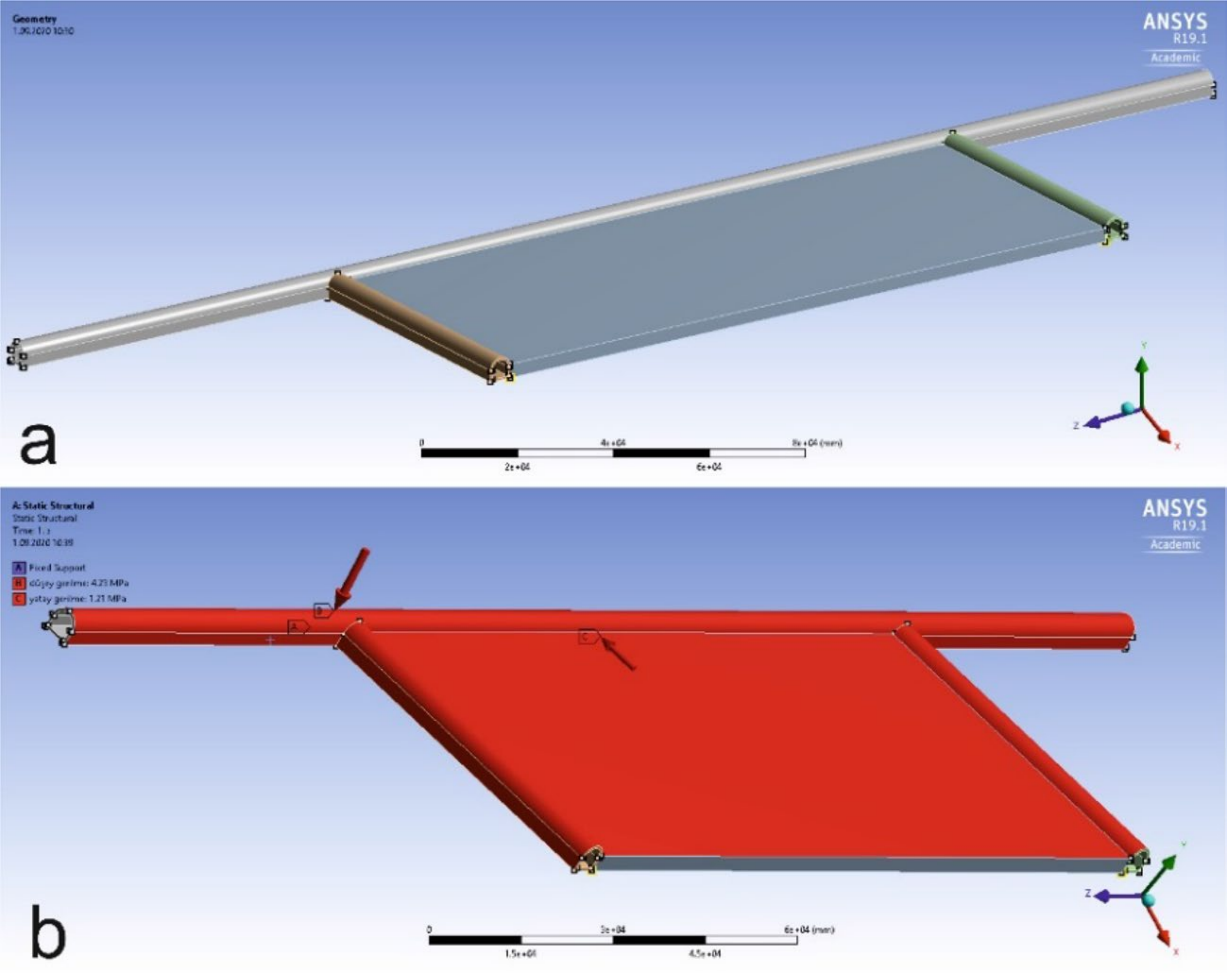
(**a**) View of the created model in ANSYS software, (**b**) application of horizontal and vertical in-situ stresses (MPa) on the model^41^.

Arch yielding supports have not been modeled in the created models within the scope of the study. During modeling studies, each model consists of 4 zones: main road, tailgate, main gate, and panel. The rock mass data given in Table 3 are entered into the zones created in each model. Mass data of Marl are entered in the main road, tailgate and main gate, while mass data of Coal in the panel zone. The site is represented on the model in the most accurate way. These processes are followed by the stress analysis phase. Reference^41^ is used in stress analysis. Figure 6a shows the view of the models in ANSYS software.

Before the stress analysis, some operations should be performed on the model. These operations include fixing the model, identification of horizontal and vertical in-situ stresses on the model, and meshing process. Horizontal and vertical in-situ stresses calculated using Eqs. (7) and (8) are applied on models in the ANSYS software. In other words, the created models are exposed to loads under the calculated in-situ stresses. Figure 6b shows the stresses applied on the main haulage road, tailgate-main gate and panel in models. Considering that even the smallest change in horizontal and vertical in-situ stresses will greatly change the stresses occurring around underground structures. This stage becomes the most important stage of the modeling process. In order for the models created to give the most accurate result, it is of great importance that the horizontal and vertical in-situ stresses in the field are calculated in the most accurate way and entered into the models. When evaluated from this point of view, the horizontal and vertical in-situ stresses acting on main haulage road, tailgate-main gate and panel in the models should be applied to these regions considering the conditions in which the longwall model created is just underground. The force vectors shown in Fig. 6b represent the calculated horizontal and vertical in-situ stresses. These stresses on the relevant regions are defined with the help of the force vectors as seen in the Fig. 6.

**Figure 7:**
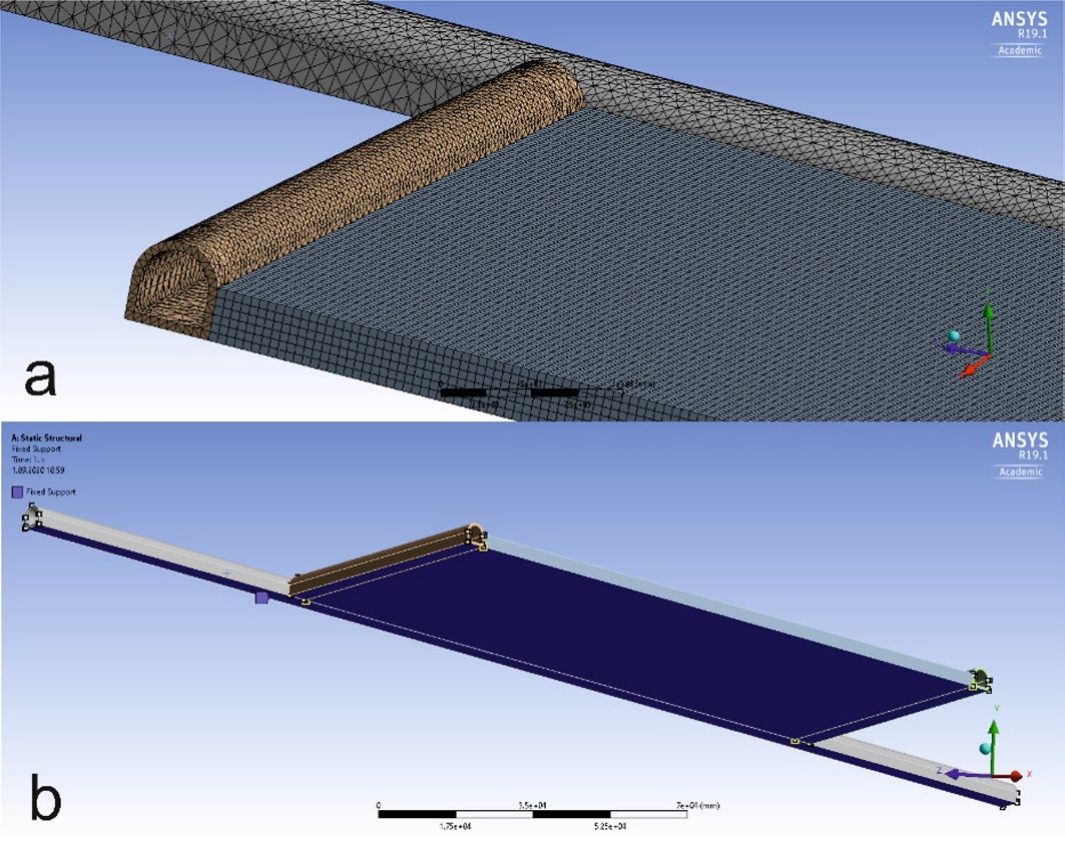
(**a**) View of the model after the meshing process, (**b**) fixing the model^41^.

The view of the model after the meshing process is given in Fig. 7a. The aim of the meshing process on the model is to divide the model into small parts, allowing more precise analysis. Another step before the stress analysis is to fix the model in the opposite direction of the load or stress in order to resist the applied stress and loads. Figure 7b shows the fixed version of the model.

The force vector shown in Fig. 7 is the fixing model used to fix the created model on a ground similar to real life case.

## Results and evaluation

In calculations, the Von Mises stress criterion is employed to determine the principal stress value. It is expressed by the formula^42^.

9 $${\sigma }_{vm}=\sqrt{3}.J$$

where J is given by:

10 $$J =\frac{1}{\sqrt{6}}.\sqrt{({\sigma }_{1}-{\sigma }_{2}{)}^{2}+({\sigma }_{2}-{\sigma }_{3}{)}^{2}+({\sigma }_{3}-{\sigma }_{1}{)}^{2}}$$

where ${\sigma }_{1}$: major principal stress; ${\sigma }_{2}$: intermediate principal stress; ${\sigma }_{3}$: minor principal stress.

**Figure 8:**
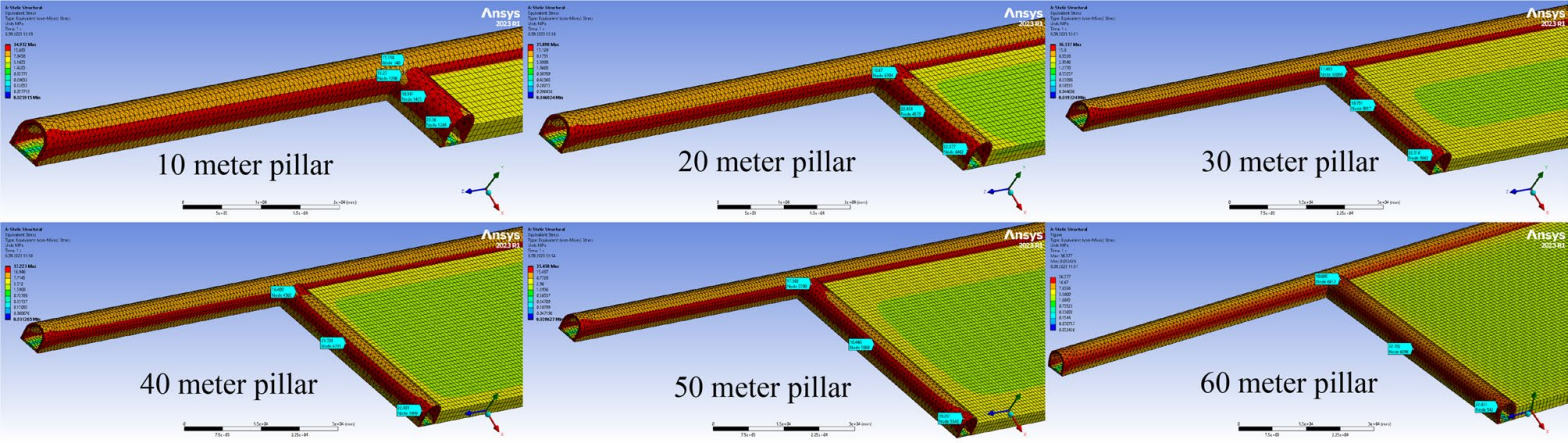
Stress distributions in different pillar distances^41^.

Following the determination of the load conditions on the created models, the models are run, and the occurred stress values are calculated. Stress distributions in different pillar distances are given in Fig. 8.

**Figure 9:**
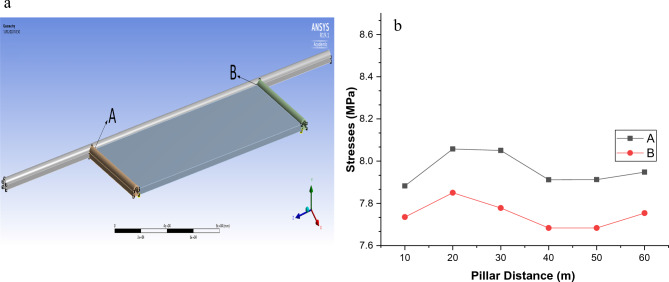
(**a**) Measurement zones on the main road^41^. (**b**) The pillar distance-based stresses occurred in the main road.

The junction points of the main road and tailgate and main gate are identified, and the stress values occurring in these zones are calculated. Figure 9a shows the measurement points on the main road. In Table 4, the stresses occurring depending on the pillar distances at the determined measurement points are given, while Fig. 9b gives a graphical display of these stresses.

**Table 4:** Vertical stresses occurred in the main road at different pillar distances.

Stresses occurred in the main road (MPa)		
Pillar distance	Zone A	Zone B
60	7.9478	7.7540
50	7.9128	7.6835
40	7.9120	7.6835
30	8.0506	7.7779
20	8.0574	7.8503
10	7.8828	7.7349

**Figure 10:**
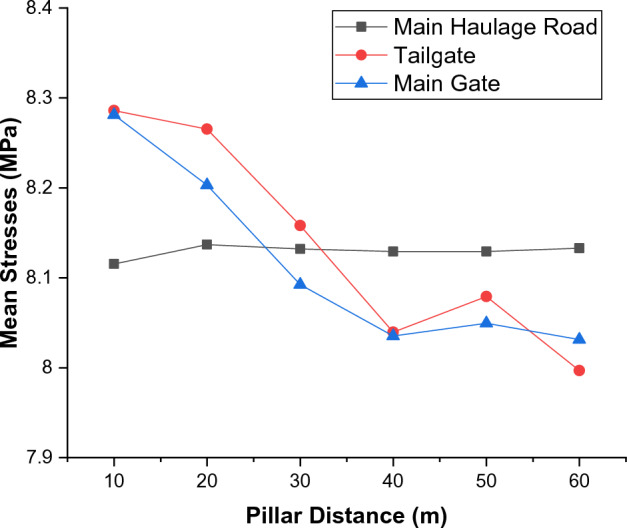
Mean vertical stresses occurred in the main road, tailgate and main gate at different pillar distances.

Considering the pillar distance-based stresses occurring in the main road, which are given in Table 4 and Fig. 9b; it is seen that these stress values are very close to each other. It is observed that the lowest stress that occurred in zone A in the main road is 7.8828 MPa at a pillar distance of 10 m, while there is a 0.82% decrease compared to the stress value at a pillar distance of 60 m. On the other hand, it is observed that the lowest stress that occurred in zone B is 7.6835 MPa at a pillar distance of 40 m, while there is a 0.91% decrease compared to the stress value at a pillar distance of 60 m. Following these zone-based stress values, in order to evaluate the stress distribution on the main road, tailgate and main gate more objectively by the pillar distance, mean stress values occurring along these zones are calculated. The mean stress values calculated are given in Table 5. The stresses occurring along the main road at different pillar distances are collectively given in Table 6. Graphical display of the mean stress distributions occurring in the main road, tailgate and main gate at different pillar distances are given in Fig. 10.

**Table 5:** Mean vertical stresses occurred at different pillar distances.

Mean stresses (MPa)			
Pillar distance	Main road	Tailgate zone	Main gate zone
10	8.1155	8.2859	8.2813
20	8.1369	8.2654	8.2031
30	8.1320	8.1580	8.0924
40	8.1292	8.0396	8.0353
50	8.1292	8.0790	8.0493
60	8.1330	7.9967	8.0313

**Table 6:** Vertical stresses occurred in the main road at different pillar distances.

Distance (m)	Stresses occurred in the main road at different pilar distances (MPa)					
	60 m	50 m	40 m	30 m	20 m	10 m
0	7.9478	7.9128	7.9120	8.0506	8.0574	7.8828
3	8.2464	8.2487	8.2481	8.1905	8.2509	8.2650
6	8.1928	8.1930	8.1930	8.1703	8.1969	8.2015
9	8.1113	8.1114	8.1114	8.1011	8.1133	8.1064
13	8.1394	8.1395	8.1395	8.1344	8.1402	8.1267
16	8.0571	8.0571	8.0571	8.0550	8.0570	8.0403
19	8.2597	8.2598	8.2598	8.2588	8.2592	8.2401
22	8.0312	8.0312	8.0312	8.0309	8.0305	8.0120
25	8.2338	8.2338	8.2338	8.2337	8.2329	8.2129
28	8.0646	8.0646	8.0646	8.0646	8.0637	8.0449
31	8.2503	8.2503	8.2503	8.2503	8.2494	8.2293
34	8.2156	8.2156	8.2156	8.2156	8.2146	8.1948
38	8.2441	8.2441	8.2441	8.2441	8.2431	8.2231
41	8.1550	8.1550	8.1550	8.1550	8.1541	8.1347
44	8.1772	8.1772	8.1772	8.1772	8.1763	8.1568
47	8.1075	8.1075	8.1075	8.1075	8.1066	8.0874
50	8.0674	8.0674	8.0674	8.0674	8.0665	8.0475
53	8.2418	8.2418	8.2418	8.2418	8.2408	8.2207
56	8.0674	8.0674	8.0674	8.0673	8.0665	8.0474
59	8.1081	8.1081	8.1081	8.1080	8.1072	8.0878
63	8.1148	8.1148	8.1148	8.1148	8.1139	8.0948
66	8.1439	8.1439	8.1439	8.1439	8.1430	8.1236
69	8.2398	8.2398	8.2398	8.2398	8.2389	8.2187
72	8.0889	8.0889	8.0889	8.0889	8.0880	8.0689
75	8.0999	8.0999	8.0999	8.0998	8.0990	8.0799
78	8.2296	8.2296	8.2296	8.2295	8.2286	8.2089
81	8.0826	8.0826	8.0826	8.0825	8.0817	8.0624
84	8.1922	8.1922	8.1922	8.1922	8.1913	8.1715
88	8.1186	8.1186	8.1186	8.1186	8.1177	8.0983
91	8.1460	8.1460	8.1460	8.1459	8.1451	8.1257
94	8.0501	8.0501	8.0501	8.0500	8.0492	8.0303
97	8.1002	8.1002	8.1002	8.1001	8.0993	8.0798
100	8.1765	8.1765	8.1765	8.1765	8.1756	8.1559
103	8.2308	8.2308	8.2308	8.2307	8.2298	8.2099
106	8.2395	8.2395	8.2395	8.2395	8.2386	8.2187
109	8.2215	8.2215	8.2215	8.2214	8.2205	8.2005
113	8.1535	8.1535	8.1535	8.1534	8.1525	8.1330
116	8.1484	8.1484	8.1484	8.1484	8.1475	8.1281
119	8.1236	8.1236	8.1236	8.1236	8.1227	8.1032
122	8.2665	8.2665	8.2665	8.2665	8.2656	8.2457
125	7.9983	7.9982	7.9982	7.9982	7.9975	7.9791
128	8.0748	8.0745	8.0746	8.0745	8.0741	8.0552
131	8.0569	8.0562	8.0562	8.0562	8.0564	8.0385
134	8.1811	8.1790	8.1791	8.1792	8.1810	8.1640
138	8.1761	8.1714	8.1714	8.1716	8.1767	8.1630
141	8.1122	8.1025	8.1026	8.1030	8.1140	8.1070
144	8.0264	8.0075	8.0078	8.0082	8.0297	8.0331
147	8.0502	8.0066	8.0072	8.0076	8.0545	8.0633
150	7.7540	7.6835	7.6835	7.7779	7.8503	7.7349
Mean	8.1330	8.1292	8.1292	8.1320	8.1369	8.1155

Considering the mean stress values given in Tables 5, 6 and Fig. 10, it is seen that the stresses occurring along the main road at different pillar distances are quite close to each other. It is seen that the lowest mean stresses in the main road occur at a pillar distance of 10 m with 8.1155 MPa, while the highest mean stresses at a pillar distance of 20 m with 8.1369 MPa. Within these stresses, there is only a 0.26% stress difference between the maximum value and the minimum value. This stress change is not thought to have any negative impact on the stability of the main road. Examining the mean stress distribution on the tailgate, it is observed that the lowest mean stresses occur at a pillar distance of 60 m with 7.9967 MPa, while the highest mean stresses at a pillar distance of 10 m with 8.2859 MPa.

In this case, there is a 3.5% change between the minimum and maximum mean stress values occurring in the tailgate. Considering the geomechanical parameters of the formation in which the tailgate is opened, this change is not thought to have any adverse effect on the stability of the tailgate. Examining the mean stress values occurring in the main gate, it is seen that the lowest mean stresses occur at a pillar distance of 60 m with 8.0313 MPa, while the highest mean stresses at a pillar distance of 10 m with 8.2813 MPa. It is seen that there is a 3.02% change between the minimum and maximum mean stress values occurring in the main gate zone. Considering the geomechanical parameters in the formation in which the main gate is opened, this change is not thought to have any adverse effect on the stability of the main gate. When evaluated in general, it is observed that the stresses occurring in the tailgate and main gate increase as the pillar distance decreases. However, it is clear that these increases in stress distribution will not have any negative effect on the stability of the main road, tailgate and main gate. It is observed that the mean stresses occurring in the main road, on the other hand, change without much dependence on the pillar distances.

When looking at the graph provided in Fig. 10, it can be observed that as the pillar distance shortens, there is an increase in stresses occurring in the tailgate and main gate, while the stresses occurring in the main haulage road remain almost unchanged. Although there may not be a noticeable difference in stress changes on the graph, it can be seen that the average stress change in the tailgate from a 60-m pillar distance to a 10-m pillar distance is 0.2892 MPa. This value is approximately equal to 30.11 tons/m^2^. In this regard, it can be concluded that as the pillar distance shortens, an additional load of 30.11 tons per square meter is applied to the tailgate. The same situation applies to the main gate, where the average stress change from a 60-m pillar distance to a 10-m pillar distance is 0.25 MPa. This value is approximately equal to 26.03 tons/m^2^. Therefore, it can be deduced that as the pillar distance decreases, an additional load of 26.03 tons per square meter is imposed on the main gate. These values indicate that the models designed within the scope of the study are in good agreement with the longwall stress mechanism.

The stress change occurring in the main haulage road region, on the other hand, decreases slightly as the pillar distance shortens. In this region, it has been observed that the average stress change is 0.0175 MPa lower compared to a 60-m pillar distance. This result is believed to be due to the increase in stress, as the pillar distance shortens, being transferred to the tailgate and main gate in accordance with the pressure arch theory^43,44^. Additionally, parallel to the conducted study, “Ref.^12^ studied 6 different panel pillar distances. They have examined the stress conditions occurring at different pillar sizes based on the safety factor and indicated that, through detailed numerical modeling studies, the pillar size can be reduced, allowing for the determination of an optimal distance.” Similarly, in parallel with the conducted study, in their research, “Ref.^14^ evaluated the stresses occurring at the pillars using a numerical analysis method. Upon examining the obtained results, it is observed that the results align with those achieved within the scope of the study.”

Besides, considering the coal recovery rates calculated according to the longwall dimensions and pillar distances designed in the models in case the pillar distance is reduced to 10 m the available coal recovery rate will increase to 83.33% due to the decrease in panel pillar.

## Conclusion

In longwall mining, the stability of main roads is crucial for production, support, ventilation, and safe production throughout the mine life. On the other hand, to work by minimizing production losses considering work safety is important for the efficient production of underground sources. In the study, pillar distances at the end of the panel are investigated for various pillar distances in order to prevent damage to the main roads, safe transportation of the longwall equipment to the new panel and the continuity of mine production. In the stress analysis, the lowest stress occurring on the main road at a pillar distance of 10 m is calculated as 8.1155 MPa. The stress value at a pillar distance of 60 m is calculated as 8.1330 MPa. In this context, considering the strength parameters of the rocks surrounding the main road, it is found that the pillar distance of 10 m does not cause a problem in the main road stresses. Furthermore, coal recovery will be increased compared to other pillars distances. Finally, it should be stated that in modeling pillar size studies, work safety should be especially considered.

## Data Availability

The datasets generated during and/or analyzed during the current study are available from the corresponding author on reasonable request.

## References

[1] Hartman, H. L. & Mutmansky, J. M. *Introductory Mining Engineering*. *Introductory Mining Engineering* (2002).

[2] Peng SS, Chiang HS (1984). Longwall mining.

[3] Hebblewhite, B. K. Future trends for underground thick seam coal mining in Australia. in *Australasian Institute of Mining and Metallurgy Publication Series* (2000).

[4] Özfırat, M., Şimşir, F. & Gonen, A. A Brief Comparison of Longwall Methods Used at Mining of Thick Coal Seams. *Proc. 19th Int. Min. Congr. Fair Turk. IMCET Izmir Turk.* (2005).

[5] Yasitli NE, Unver B (2005). 3D numerical modeling of longwall mining with top-coal caving. Int. J. Rock Mech. Min. Sci..

[6] Vakili A, Hebblewhite BK (2010). A new cavability assessment criterion for longwall top coal caving. Int. J. Rock Mech. Min. Sci..

[7] Shabanimashcool M, Li CC (2012). Numerical modelling of longwall mining and stability analysis of the gates in a coal mine. Int. J. Rock Mech. Min. Sci..

[8] Suchowerska AM, Carter JP, Merifield RS (2014). Horizontal stress under supercritical longwall panels. Int. J. Rock Mech. Min. Sci..

[9] Zhu D, Chen Z, Du W, Zhang L, Zhou Z (2018). Caving mechanisms of loose top-coal in longwall top-coal caving mining based on stochastic medium theory. Arab. J. Geosci..

[10] Wang S, Li X, Wang S (2018). Three-dimensional mineral grade distribution modelling and longwall mining of an underground bauxite seam. Int. J. Rock Mech. Min. Sci..

[11] Le TD, Oh J, Hebblewhite B, Zhang C, Mitra R (2018). A discontinuum modelling approach for investigation of Longwall Top Coal Caving mechanisms. Int. J. Rock Mech. Min. Sci..

[12] Tewari S, Kushwaha A, Bhattacharjee R, Porathur JL (2018). Crown pillar design in highly dipping coal seam. Int. J. Rock Mech. Min. Sci..

[13] Kang H, Wu L, Gao F, Lv H, Li J (2019). Field study on the load transfer mechanics associated with longwall coal retreat mining. Int. J. Rock Mech. Min. Sci..

[14] Sinha S, Walton G (2019). Investigation of longwall headgate stress distribution with an emphasis on pillar behavior. Int. J. Rock Mech. Min. Sci..

[15] Islavath SR, Deb D, Kumar H (2020). Development of a roof-to-floor convergence index for longwall face using combined finite element modelling and statistical approach. Int. J. Rock Mech. Min. Sci..

[16] Alehossein H, Poulsen BA (2010). Stress analysis of longwall top coal caving. Int. J. Rock Mech. Min. Sci..

[17] Gonen, A. & Kose, H. Stability analysis of open stopes and backfill in longhole stoping method for asikoy underground copper mine. *Arch. Min. Sci.* (2011).

[18] Kun M, Onargan T (2013). Influence of the fault zone in shallow tunneling: A case study of Izmir Metro Tunnel. Tunn. Undergr. Space Technol..

[19] Woo KS, Eberhardt E, Elmo D, Stead D (2013). Empirical investigation and characterization of surface subsidence related to block cave mining. Int. J. Rock Mech. Min. Sci..

[20] Wang J, Yang S, Li Y, Wei L, Liu H (2014). Caving mechanisms of loose top-coal in longwall top-coal caving mining method. Int. J. Rock Mech. Min. Sci..

[21] Basarir H, Ferid Oge I, Aydin O (2015). Prediction of the stresses around main and tail gates during top coal caving by 3D numerical analysis. Int. J. Rock Mech. Min. Sci..

[22] Jiang L (2016). Influence of fracture-induced weakening on coal mine gateroad stability. Int. J. Rock Mech. Min. Sci..

[23] Mandal PK (2018). Assessment of roof convergence during driving roadways in underground coal mines by continuous miner. Int. J. Rock Mech. Min. Sci..

[24] Zhu S, Feng Y, Jiang F, Liu J (2018). Mechanism and risk assessment of overall-instability-induced rockbursts in deep island longwall panels. Int. J. Rock Mech. Min. Sci..

[25] Klishin VI, Fryanov VN, Pavlova LD, Opruk GY (2019). Modeling top coal disintegration in thick seams in longwall top coal caving. J. Min. Sci..

[26] Yetkin ME, Şimşir F (2019). Determination of most suitable working height of powered roof support considering roof stresses. J. Min. Sci..

[27] Zhang Q, Yue J, Liu C, Feng C, Li H (2019). Study of automated top-coal caving in extra-thick coal seams using the continuum-discontinuum element method. Int. J. Rock Mech. Min. Sci..

[28] Zhang X, Gong P, Wang K, Li J, Jiang Y (2019). Characteristic and mechanism of roof fracture ahead of the face in an LTCC panel when passing an abandoned roadway: A case study from the Shenghua coal mine. China. Rock Mech. Rock Eng..

[29] Weijie W, Shengli Y, Meng L, Jinwang Z, Chuanbo W (2022). Motion mechanisms for top coal and gangue blocks in longwall top coal caving (LTCC) with an extra-thick seam. Rock Mech. Rock Eng..

[30] Deng G (2023). Fracture mechanisms of competent overburden under high stress conditions: A case study. Rock Mech. Rock Eng..

[31] Skrzypkowski, K. Decreasing mining losses for the room and pillar method by replacing the ınter-room pillars by the construction of wooden cribs filled with waste rocks. *Energies* **13**, (2020).

[32] Skrzypkowski, K. Comparative analysis of the mining cribs models filled with gangue. *Energies* **13**, (2020).

[33] Hoek E, Brown ET (1997). Practical estimates of rock mass strength. Int. J. Rock Mech. Min. Sci..

[34] Sheorey PR (1994). A theory for In Situ stresses in isotropic and transverseley isotropic rock. Int. J. Rock Mech. Min. Sci..

[35] *SpaceClaim. 3D Modeling Software. (2022). Release 22.2. *https://www.ansys.com/products/3d-design/ansys-spaceclaim.

[36] RocData. *Rock, Soil and Discontinuity Strength Analysis, Version 5.0*. (2014).

[37] Verma AK, Deb D (2013). Numerical analysis of an interaction between hydraulic-powered support and surrounding rock strata. Int. J. Geomech..

[38] Yetkin ME, Simsir F, Ozfirat MK, Ozfirat PM, Yenice H (2016). A fuzzy approach to selecting roof supports in longwall mining. S. Afr. J. Ind. Eng..

[39] Destanoglu, N., Taskin, F. B., Tastepe, M. & Ogretmen, S. *Omerler Mechanized Longwall Application*. (Turkish Coal Administration, (in Turkish)., Ankara, 2000).

[40] Ozfirat, M. K. Investigations on Determining and Decreasing the Coal Loss At Fully-Mechanized Production in Omerler Underground Coal Mine. (PhD Thesis, Institute of Natural and Applied Sciences, Dokuz Eylul University. Izmir (in Turkish)., 2007).

[41] *Ansys. Ansys Workbench. (2022).Release 22.2. *https://www.ansys.com/products/structures/ansys-mechanical.

[42] Rocscience Inc 8. Rocscience. (2014).

[43] Poulsen BA (2010). Coal pillar load calculation by pressure arch theory and near field extraction ratio. Int. J. Rock Mech. Min. Sci..

[44] Wang S-L (2016). Numerical investigation of coal pillar failure under simultaneous static and dynamic loading. Int. J. Rock Mech. Min. Sci..

